# Sources of knowledge empowerment amongst pregnant women with TB disease: A qualitative study in South Africa

**DOI:** 10.1111/hex.13947

**Published:** 2023-12-26

**Authors:** Mulondo Seani Adrinah, Khoza Lunic Base, Lebese Rachel Tsakani

**Affiliations:** ^1^ Department of Advanced Nursing Science Faculty of Health Sciences, The University of Venda Thohoyandou South Africa; ^2^ Department of Health Studies The University of South Africa Tzaneen South Africa

**Keywords:** empowerment, healthcare workers, knowledge, pregnant women, tuberculosis disease

## Abstract

**Background:**

Providing relevant knowledge to empower all pregnant women diagnosed and nondiagnosed with tuberculosis (TB) is essential worldwide. Proper dissemination of health information for pregnant women could assist in preventing TB complications amongst women and babies. The study aimed to describe the sources of knowledge that empower pregnant women diagnosed with TB and improve their quality of life in Limpopo Province, South Africa.

**Methodology:**

The study followed a qualitative, exploratory and descriptive research design. The study was conducted in 12 selected primary healthcare facilities in three crisis districts. Thirty‐five pregnant women with TB disease were purposively selected, and face‐to‐face interviews were conducted to generate data, which were analysed using the thematic approach. Measures to ensure trustworthiness and ethical standards were adhered to.

**Results:**

The findings of this study revealed that healthcare workers, community stakeholders, and TB ambassadors are the primary sources of knowledge dissemination for capacitating women diagnosed with TB.

**Conclusion:**

Most pregnant women lacked knowledge regarding TB ambassadors as sources of information for empowering women, community awareness campaigns, and Google searches as sources of information sharing. All stakeholders need to work together, considering the patients' charter for TB care that sets out the right for respect and information sharing. The emphasis of this study was on developing a comprehensive educational intervention that could assist in improving the quality of TB services offered to pregnant women.

## 1 INTRODUCTION

Providing relevant knowledge to empower all pregnant women, diagnosed and nondiagnosed with tuberculosis (TB), is essential worldwide.^1^ Empowerment is a multidimensional process through which women gain power and control over their sexual and reproductive health.^2^ Pregnant women with knowledge have confidence and self‐efficacy, which helps them make choices and have positive behavioural change that improves adequate utilisation of Antenatal care services (ANCs).

Midwives usually provide high‐quality prepregnancy care, prenatal care and postnatal care at primary healthcare (PHC) facilities, community healthcare centers (CHCs), and hospitals to improve pregnancy outcomes.^3^, ^4^ Family and community member support and referrals by traditional healers in African countries could positively or negatively contribute to pregnancy outcomes.^5^, ^6^ Despite the distribution of healthcare facilities throughout South Africa, access by most pregnant women is a challenge as they live in deep rural areas, in a state of poverty and poor road structures.^7^ Midwives provide health education during antenatal visits that aid pregnancy outcomes.^8^ However, women need other sources of health information, such as radio stations, televisions and the Internet, to supplement the education received from midwives.^3^, ^9^

TB is a leading cause of morbidity and mortality worldwide. The World Health Organisation (WHO)^10^ indicated an increase in TB deaths from 1.4 to 1.5 million amongst women of childbearing age 15–49 years. Delays in screening, diagnosis and commencement of TB treatment could be associated with cultural issues and knowledge deficiency due to inadequate dissemination of essential TB information to women.^11^ The knowledge deficiency of midwives on routine screening for TB contributes to late diagnosis that aggravates the progression of TB, leading to TB complications for both mother and baby.^10^ The outbreak of the COVID‐19 pandemic has reduced access to PHC facilities by most pregnant women worldwide.^12^ The pandemic interrupted the functioning of most PHC facilities, decreased TB screening and testing rates and also affected the dissemination of health‐related matters to pregnant women with TB.

In South Africa, the overall incidence of TB death is 61,000. Women of childbearing age could be amongst the mortality cases of TB. However, accurate reporting of TB statistics amongst childbearing women in South Africa has been challenging since 1990.^13^, ^14^ Studies were done on associations between women's mandate and some aspects, such as fertility and contraception. There are insufficient studies regarding women's empowerment and pregnancy, including maternal TB.^15^ Proper dissemination of health information to encourage pregnant women with TB disease, using various sources, could assist in preventing TB complications amongst women and babies. This study describes the sources of knowledge to empower pregnant women diagnosed with TB and on a four‐drug regimen to improve their quality of life in Limpopo Province, South Africa.

## 2 THEORETICAL FRAMEWORK

**Figure 1:**
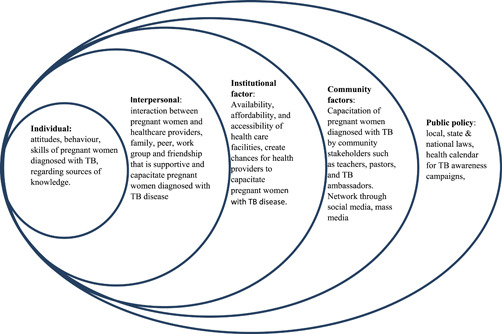
Socio‐ecological model of health promotion (adapted from McLeroy et al.^16^). TB, tuberculosis.

This study adapted the socio‐ecological model to elucidate the sources of information to empower pregnant women with TB to improve the quality of life for both mother and baby. The model comprises individual, interpersonal, community, organisational and societal factors to promote health as presented in Figure 1, adapted from McLeroy et al.^16^ Researchers obtained no consent from the developers of the socio‐ecological model as it was adapted to suit the situation under study.

### 2.1 Individual factors

Pregnant women with TB possess knowledge, attitudes, behaviour, self‐efficacy, resiliency and skill. They can make positive decisions and access PHC facilities for ANCs for early TB screening, diagnosis and treatment to improve their well‐being.

### 2.2 Interpersonal factors

Interpersonal factors are the social support systems surrounding women, which include the family, peer, workgroup and friendship networks interacting with TB pregnant women. They are sources of knowledge for capacitation and supporting women in treatment adherence to prevent TB complications.

### 2.3 Institutional factors

Availability, affordability and accessibility of PHC facilities create opportunities for health providers to capacitate pregnant women with TB. However, long waits in queues and long travel distances by foot discourage women with TB from utilising ANCs adequately. Adequate utilisation improves the well‐being of both mother and baby.

### 2.4 Community factors

Community stakeholders such as teachers, pastors and TB ambassadors are sources of knowledge for capacitating pregnant women with TB. Information dissemination can be carried out during community mass meetings. Social and mass media networks can reach many women with TB to increase awareness and promote the health status of both mother and baby.

### 2.5 Public policy

TB and pregnancy week awareness campaigns may focus on adherence to TB treatment at PHC facilities. Consider the local, state and national laws and health calendars for TB awareness campaigns to reach all pregnant women with TB.

## 3 MATERIALS AND METHODS

### 3.1 Research design

The study followed a qualitative, exploratory and descriptive research design. The researchers needed to explore in‐depth, gain insight and better understand the nature of sources of knowledge dissemination to empower women, thereby improving the quality of life of pregnant women with TB. Data were obtained as descriptive narratives from the research participants.^17^

### 3.2 Study setting

The study was conducted at selected PHC facilities in Limpopo Province. The province comprises—5 districts, 44 hospitals, 37 CHCs and 404 PHC facilities. PHC facilities within three crisis districts' were selected due to their high prevalence and incidence of TB. Most people diagnosed with TB, including pregnant women, are managed at PHC facilities.

### 3.3 Population, sample and sampling

The target population for this study was 60 pregnant women, which was a summation of 3–6 from 12 selected PHC facilities in three districts. The statistics of diagnosed pregnant women with TB differ from one PHC facility to another, and are lower than other population groups. The accessible population for this study was 35 pregnant women who agreed to be part of the study. Researchers used a purposive, nonprobability and convenient sampling approach to select 35 participants.^18^ Facilities were selected using a purposive sampling approach based on the prevalence of TB statistics amongst pregnant women. The selected participants represented the desired population as they were available in the PHC facility during data generation and were on TB treatment.^18^ The participants were in the age bracket of 20–42 years.

### 3.4 Data collection and instrument

Researchers were females and lecturers with PhD degrees with vast experience in research. The researchers developed a semistructured interview schedule to collect participant data.^19^ The interview questions were formulated in English based on the study topic and purpose. Questions were translated into Tshivenda, Sepedi, and Xitsonga by the first, second and third authors, respectively, to accommodate the participants' mother tongues. The third researcher was an expert in qualitative research. Researchers made the appointment through the TB coordinator of each PHC facility; 2–4 pregnant women with TB agreed to be part of the study. The purpose of the study was explained to participants, after which they signed consent forms to be part of the study and to allow the use of a voice recorder. Participation was voluntary, and no remuneration was given to the participants.

Researchers sought demographic data, such as age, area of residence and time of first ANCs. The in‐depth interviews were conducted with the participants in face‐to‐face sessions in the private unit offered by the operational manager of PHC facility. The following questions were asked: ‘What do you think are the sources of knowledge to empower pregnant women with TB to improve their quality of life?’ Second, ‘Share with me what you think are the sources of information required by pregnant women to prevent TB complications’. All responses were followed by probing to get more detailed information from participants.

A pretest was conducted on 10% of the total participants. Three participants were interviewed to evaluate the legibility of the research questions and to rephrase them where necessary. Participants were not part of the main study. The first researcher introduced the subject to the participants, making them comfortable and establishing rapport and trust. The researchers and participants were not related. Thirty‐five participants were interviewed, and none refused. The researchers collected in‐depth, detailed, complex and multiple information and determined to have reached data saturation when no longer hearing something new from participant 25. Enough data were collected to draw the necessary conclusion. However, despite the nonproduction of value‐added insights, the sessions continued until 35 participants from the 12 selected PHC facilities had been interviewed. The researchers' interest in covering 12 selected PHC facilities permitted the study to be conducted. The researchers recorded and documented interview sessions in the field notes. The interview sessions lasted 45–60 min each and were not repeated. Member checking was done on participants to ensure that the recorded and transcribed information was correct.^20^ Three researchers managed to reach 20–25 the participants.

### 3.5 Data analysis

The data analysis was conducted using the thematic approach and proceeded along the recommended steps 1–5 of Creswell and Poth^21^ and Creswell and Creswell.^22^ Therefore, three researchers familiarised themselves with the data by transcribing through reading the field notes and listening to audio‐recorded interviews to gain an understanding in step 1. In step 2, researchers generated initial codes by highlighting phrases or sentences and creating ‘codes’ to describe their content. In step 3, researchers developed themes by coming up with themes that combine several codes into a single theme. Step 4 is reviewing themes by returning to the data set and comparing the themes against collected data to ensure that themes are useful and accurately represent data. Step 5 is defining and naming themes by creating a succinct and easily understandable name for each theme and emerging subthemes. Researchers went through these steps with the guidance of the third researcher, an expert in qualitative research. The third researcher verified transcripts as an independent coder.

### 3.6 Trustworthiness

Credibility, dependability, confirmability and transferability, as Lincoln and Guba's^23^ four principles for scientific research, were recommended. Rapport and trust were developed with the participants to establish credibility. The researchers achieved the dependability of the data by using a voice recorder and field notes to verify correctness. Confirmability was guaranteed by recording the participants' real responses through triangulation using a voice recorder and field notes. Researchers achieved transferability by clearly describing the research methodology to ensure its applicability in other study contexts.

## 4 RESULTS

The study's findings are based on in‐depth interviews conducted with 35 participants. The demographic profile revealed that most participants were 20–30 years old, and very few were 41 years and above. Most were from rural areas. Most reported late for ANCs and failed to meet the WHO recommendations of at least four ANC visits.^7^ The findings of this study focused on TB and sources of knowledge empowerment to improve maternal and neonatal health. Although 35 participants were interviewed, not all the data presented to avoid repeating similar quotes, which could unnecessarily increase the volume. One quote was given to participants who expressed similar information. Two themes based on the study purpose and five subthemes that emerged from data analysis are summarised in Table 1.

**Table 1:** Themes and subthemes.

Themes	Subthemes
Theme 1: Participants' expectations of the healthcare workers who should capacitate pregnant women with TB.	1.1 Healthcare workers are sources to capacitate pregnant women with TB.
	1.2 Community stakeholders and TB ambassadors are sources to capacitate pregnant women with TB.
	1.3 Integration of community, family, traditional healers, and healthcare workers in capacitating pregnant women with TB.
Theme 2: Participants' comments on sources of information to prevent TB complications.	2.1 Community awareness campaigns for pregnant women, diagnosed and nondiagnosed with TB.
	2.2 Information sharing through social media, cell phones, Google searches and radio stations.

### 4.1 Theme 1: Participants' expectations of healthcare workers as sources to capacitate pregnant women with TB

Most participants indicated that healthcare workers play a crucial role in capacitating women during pregnancy, labour and the puerperium period by delivering health education to promote maternal and child well‐being. Late presentation at ANCs was a significant challenge as most women could be disadvantaged from the benefits of antenatal education. The following three subthemes emerged:

#### 4.1.1 Subtheme 1.1: Healthcare workers are sources to capacitate pregnant women with TB

Midwives remain critical sources of health information for maternity care services worldwide, although most fail to perform routine TB screening during ANCs.^24^ Most participants expressed healthcare workers as essential sources to empower pregnant women with TB. One participant said:

The nurses and doctors used to give health education when we go for ANCs (Pause), but since the COVID‐19 pandemic, nurses no longer give health education to groups of pregnant women. (P9)

The interruption of the functioning of PHC facilities was affected by the outbreak of COVID‐19. In supporting that, another participant similarly said:

Yaaa, health education used to be given to all people—mothers who have brought children for immunisation, elderly people, pregnant women during booking. (Pause); however, since COVID‐19, nurses no longer have time with us! We are checked and given treatment refills! (P16)

Participants expressed that the home‐based care providers do not serve the community as expected. Home‐based Care providers were trained to reach people in need at the community level, including pregnant women with TB. Contrary to their expected role, one participant said:

I see the home‐based care providers walking along the streets, but they do not enter our household and give health education or advice…. They enter where there is a bedridden person and meet them at the clinic for treatment refills!! (P6)

Home‐based care providers have no secrecy, respect and have negative attitude towards women with TB. (P28)

#### 4.1.2 Subtheme 1.2: Community stakeholders and TB ambassadors are sources to capacitate pregnant women with information on TB

Healthcare workers established relationships with community stakeholders, such as traditional leaders, to attain common goals and health for community members, including pregnant women with TB. Participants indicated that stakeholders are well‐known, respected and accepted by community members as sources of knowledge in promoting health.

One of the participants said:

If community stakeholders, such as pastors, are educated on TB information, messages on preventive measures and treatment adherence can be conveyed to all community members during Sunday services. (P1)

Another participant said:

All women diagnosed and non‐diagnosed with TB need information about TB (Pause); I think it may be helpful if teachers, traditional leaders, and others who are well‐known in the community are educated on TB matters so that they can impart to the community members. (P20)

While still on that issue, another participant said:

Traditional leaders may also share during formal community meetings held once a month! I hope it will assist in stopping this stranger called ‘TB’ and reduce the TB stigma! (P25)

Another participant expressed similar ideas:

Teachers can partner with the nurses who visit our children at schools. They may continue giving advice and health education to learners, especially in secondary schools where most learners are teenagers! (P7)

In response to that, another participant said:

Yoo! TB does not exclude! Even teenagers may become pregnant and infected with TB. (P29)

Regarding the availability of TB ambassadors in the community, most participants were not knowledgeable about these people. One participant said:

In our village, two people (a woman and a man) have disclosed TB disease. During community gatherings called by the chief and TB awareness campaigns, they are given a slot to tell that TB is curable and we can adhere to treatment! (Pause) Yaaa, to be honest with you, it is beneficial, although some people may talk badly behind their backs! (P4)

#### 4.1.3 Subtheme 1.3: Integration of community, family members, traditional healers and healthcare workers to capacitate pregnant women with TB information

Family and community members, stakeholders and healthcare workers are the primary sources of knowledge in the community on health promotion and education focusing on treatment adherence. Most participants indicated that the effective integration of these members could play a significant role in capacitating pregnant women with TB information to improve their quality of life. One participant said:

You know, community stakeholders can adopt that slogan of ‘Working together, we can make it’. It is good to involve people when planning such community initiatives. (P3)

Another participant said:

People from different areas can share their ideas, rules, and health‐related information during community meetings to promote better health status and prevent TB complications. (P23)

### 4.2 Theme 2: Participants' comments on sources of information to prevent TB complications

Community members need awareness about sources of health information to improve health. Participants acknowledged various health information sources under the following subthemes.

#### 4.2.1 Subtheme 2.1: Community awareness campaigns for pregnant women, diagnosed and nondiagnosed with TB

In Limpopo Province, healthcare workers and home‐based care providers conduct health awareness campaigns through the Department of Health to increase awareness amongst pregnant women and other community members. A few participants expressed participation in TB awareness campaigns, pregnancy‐week, and Cancer week campaigns. Most participants were not conversant with campaigns. One of the participants said:

I know of activities conducted in our village, hmmmmm…. We have community recreational facilities where people have gym activities, projects for creche, and sowing. (P33)

Another participant said:

I know that mobile clinics are offered once a month, but since COVID‐19, they are no longer coming! (Pause). That is a challenge for some of us who cannot pay for transport to the clinic because it's too far!! (P18)

Amongst the few participants who are knowledgeable about campaigns, one said:

During the TB or pregnancy week campaigns, nurses give education and put posters with pictures and information about TB on the board; pregnant women can read and follow the instructions to promote their well‐being and that of the baby. (Pause) However, those who cannot read well may see the pictures! (P13)

#### 4.2.2 Subtheme 2.2: Information‐sharing through social media, cell phones, Google search, and radio stations

Social media platforms such as Facebook, LinkedIn, Twitter, WhatsApp, Google search, smart cell phones and radio stations can disseminate health‐related information that promotes well‐being and prevent TB complications. One participant said:

These days, televisions, radios, and cell phones are used as sources of health information. Most people in the villages have radios, used as main sources of information. (P35)

Another participant said:

Some of us have smartphones that can use WhatsApp and the Internet through Google search for information about TB. Social media, such as Facebook and Twitter, can also be used. I think they are beneficial (pause), … it's just unfortunate for those who do not have smartphones. (P2)

## 5 DISCUSSION

The study explored sources of knowledge empowerment amongst pregnant women in Limpopo Province. The study results indicated that healthcare providers are the primary sources of knowledge to capacitate pregnant women with TB. Midwives are the key providers of ANCs and give health education to improve early presentation at ANC centres to promote maternal and neonatal well‐being. The participating pregnant women also acknowledged that television, radio stations and other social media are sources of transmitting information to women. The socio‐ecological model, as applied in this study, clarifies that health information acquired by pregnant women with TB promotes positive attitudes and self‐efficacy and changes in behaviour that promote adequate access and utilisation of PHC facilities. Hence, healthcare providers, stakeholders and awareness campaigns as sources for capacitating pregnant women with TB information are the social system surrounding pregnant women with TB. The study findings are discussed according to the subthemes as indicated below.

### 5.1 Healthcare workers are the primary sources to capacitate pregnant women with information on TB

Healthcare workers, such as midwives and home‐based care providers, are the social system surrounding pregnant women with TB as per the model used. These providers could be essential in providing pregnant women with knowledge that might aid in early ANC visits. Pregnant women need support, health education on causes, signs and symptoms, prevention, treatment adherence and inclusive TB complications that might arise during pregnancy. However, the disruption of the smooth functioning of PHC facilities by an outbreak of COVID‐19 could have affected the dissemination of health education by midwives. That could expose pregnant women to high‐risk factors of untreated maternal TB, such as abortion and low birth weights. The COVID‐19 outbreak has negatively impacted the delivery of ANCs worldwide.^25^

A study in Nigeria reported that pregnant women empowered with knowledge produced a positive attitude towards ANCs. Women presented early for TB screening, diagnosis and commencement of TB treatment, where sputum tested positive. Complications associated with TB were prevented amongst neonates.^26^ Contrary to this, poor capacitation of pregnant women with TB‐related information has led to poor utilisation of ANCs, contributing to congenital TB amongst Ghana‐born babies.^27^

Home‐based care providers form an integral part of PHC outreach. Pregnant women need health education on TB, supervision on treatment adherence and support through home visits to promote self‐sufficiency and good health. Although they are available and good sources of information dissemination, the study findings affirmed that providers do not visit households to assist pregnant women with any health education related to TB. This is a concern as they are expected to fill a gap between PHC facilities and the community. Poor service delivery could be associated with long travel distances and lack of financial support as they receive low levels of stipends at intervals, not a total monthly payment. Reluctancy to visiting women with TB could also be associated with fear of contracting COVID‐19 and TB. That might distort relationships with women. Pregnant women could assume that home‐based care providers might talk about their illnesses with other community members and no longer have confidence. This might increase the stigma associated with TB amongst pregnant women. Motswasele‐Sikwane et al.^28^ reported that home‐based care providers experience lack of support in their communities and seniors, long distances to travel by foot, and poor financial support. In supporting this finding, home‐based care providers do not get a full salary except a stipend. Therefore, they do not have enough money to travel to individual households and convey promotive and preventive health education on common infectious diseases, such as TB.^29^ Capacitation of women with TB‐related information could improve morale, produce a positive attitude towards ANCs and reduce TB‐related mortality in many pregnant women with TB.

### 5.2 Community stakeholders and TB ambassadors provide pregnant women with information on TB disease

The findings of this study suggest that community stakeholders could play crucial roles in capacitating women during formal community meetings and Sunday services. They are the key sources surrounding pregnant women in disseminating health information amongst women with TB. Stakeholders are supported and given informal training on health‐related information. Their activities could further assist in reducing the stigma attached to TB, as family members and other community members would be able to accept and support pregnant women with TB. Poor maternal and neonatal outcomes associated with TB could be prevented. McKenna et al.^30^ reported that the burden of pregnancy‐associated TB could only be reduced by providing health information regarding maternal TB at the community level with community stakeholders in partnership with healthcare nurses at PHC facilities.

Most participating women in this study are unfamiliar with TB ambassadors, as they are not readily available in Limpopo Province. TB ambassadors are those treated for TB disease and are encouraged to send positive messages during TB awareness campaigns that TB can be cured. Despite the unfamiliarity by most participants, it is essential to encourage TB survivors to be advocates for TB disease during campaigns and traditional meetings. Dissemination of this message could promote treatment adherence by stopping mixing Western medication with traditional herbs.^31^ Christie^32^ mentions two TB survivors who continued to give support by addressing audiences on TB‐related matters during TB campaigns and community gatherings. These kinds of information‐giving sessions could assist pregnant women in taking the initial step, utilising ANCs appropriately, early screening, diagnosis and adherence to treatment to prevent TB complications. It is essential to encourage door‐to‐door community campaigns to reach most pregnant women.

### 5.3 Integration of community, family members, traditional healers and healthcare workers in the capacitation of pregnant women with information on TB

Family members, community stakeholders and healthcare workers were also revealed as the social support surrounding pregnant women. The study findings indicated that integrating stakeholders and healthcare providers could assist incapacitation by providing health education to pregnant women with TB. The integration could promote referrals from traditional healers to clinics for management to prevent TB complications.

Govender et al.^33^ reported that a multidisciplinary community of practices is needed to generate and share knowledge that could be conveyed to pregnant women with TB in Kwazulu Natal, South Africa. McKenna et al.^30^ similarly indicated that integrating community stakeholders and healthcare providers in maternal care facilitated addressing health‐related matters on pregnancy‐associated TB, reducing maternal and neonatal mortality. In the Zambezi Province, Mozambique, it is reported that the integration of various community stakeholders and healthcare workers stimulated the interest of traditional healers in providing community‐based support, education, and therapy to pregnant women with TB. This resulted in increased access to ANCs and improved treatment adherence amongst women diagnosed with TB.^34^

### 5.4 Community awareness campaigns for pregnant women, diagnosed and nondiagnosed with TB

In this study, most participants were unfamiliar with health campaigns except for activities such as entertainment during recreational sports. Few participants knew about the TB and pregnancy week campaigns. In South Africa, health awareness campaigns on TB, pregnancy, HIV and cancer are scheduled in the national health calendar per public policy and conducted in each district. Knowledge deficiency amongst pregnant women in rural areas could be a factor in the nonattendance of these campaigns. During the campaign, health education is given about lifestyle modification and promotional matters to improve the effective utilisation of ANCs to avoid TB complications.^6^, ^35^ Woldesenbet et al.^36^ indicated that pregnancy week campaigns, TB and HIV, are essential in reducing unintended and unplanned pregnancies amongst women aged 15–49. There was a considerable improvement in maternal and neonatal outcomes. Women with TB need to be empowered during health awareness campaigns to sensitise them that TB could be curable.

### 5.5 Information sharing through social media, cell phones, Google searches and radio

Globally, most pregnant women receive health‐related information through different social media platforms. The study revealed that most pregnant women knew of various sources for sharing news that could improve and change their health behaviour. Participants indicated that radio stations are the most appropriate and accessible media platform that could convey health information related to TB matters to the public. In this study, healthcare workers use three commonly spoken languages in three districts to bring health education to community members. According to local languages, the radio stations are Phalaphala FM, Motsweding FM and Munghana Lonene FM.

The study in Malawi reported that mass media was the primary source for disseminating health information to the public to improve comprehension and bring about behaviour change. The study further reported improvement in utilising maternity care services, leading to reduced maternal and neonatal morbidity.^37^ In this study, few participants could use Google searches and social media such as WhatsApp and Facebook to gain information regarding pregnancy‐associated TB. Social media could disseminate health awareness information that could be easily accessible and communicated and reach the global world to improve adequate utilisation of ANCs, promote well‐being, and prevent TB complications.

## 6 LIMITATIONS OF THE STUDY

The study focused only on pregnant women diagnosed with TB in 12 selected PHC facilities of the three selected crisis districts of Limpopo Province to understand better sources of knowledge empowerment amongst the vulnerable group at high risk. A qualitative research design was used for this study; the sample size was small and could not represent the whole of Limpopo Province. Pregnant women nondiagnosed with TB were excluded and might have brought other factors that could have influenced the knowledge gained. The excessive use of social networking such as WhatsApp and Facebook might not disseminate health information to most pregnant women due to the unavailability of smartphone sites. At times, much of the information might be false, unreliable and full of scams.

## 7 CONCLUSION

The findings of this study revealed healthcare workers, community stakeholders and TB ambassadors as the primary sources of providing knowledge to capacitate pregnant women diagnosed with TB. However, most pregnant women lack information regarding stakeholders such as ambassadors, responsibilities of home‐based care providers, community awareness campaigns, and Google searches as information sharing. The radio was expressed as an essential media for sharing comprehensive maternal health information to prevent TB complications. The outbreak of COVID‐19 could have contributed to the poor dissemination of health information, hindered the utilisation of ANCs, and aggravated the incidences of TB. Recommendations are for developing comprehensive educational interventions that could assist in improving the standard of TB services for detection, screening, and testing to improve the quality of life amongst pregnant women. All stakeholders need to work together, considering the patients' charter for TB care that sets out the right to care and information sharing.

## AUTHOR CONTRIBUTIONS

**Mulondo Seani Adrinah**: Funding acquisition; project administration; writing—original draft; methodology; writing—review and editing. **Khoza Lunic Base**: Conceptualisation; writing—review and editing; methodology; writing—original draft. **Lebese Rachel Tsakani**: Formal analysis; writing—review and editing; methodology; writing—original draft.

## CONFLICT OF INTEREST STATEMENT

The authors declare no conflict of interest.

## ETHICS STATEMENT

An Ethical Certificate (SHS/20/PDC/34/0511) was issued by the University Ethics Committee on 11 November 2020. Limpopo Department of Health, Mopani, Vhembe and Sekhukhune districts granted permission. The Limpopo Department of Health Research Committee granted permission to conduct the study at the selected PHC facilities, and the District Executive Managers allowed the researchers access to these facilities. The researchers gave adequate information regarding the purpose of the study, the procedures involved and the opportunity to ask questions to enable participants to make informed choices. Participation was voluntary, and nonparticipation was not penalised. Participants signed consent forms before data generation. Participants could be offered a referral for counseling by midwives or psychotherapy if they experience any signs of emotional distress associated with TB stigma. Counseling could assist in changing troubling emotions, thoughts and behaviour to improve the overall quality of life. Ethical issues such as anonymity, confidentiality, privacy and freedom from harm were explained and adhered to. Informed consent was obtained from all participants. Since this study involved humans, it was conducted in accordance with the international ethical standards of the Helsinki Declaration and Good Clinical Practice.

## Data Availability

The data sets analysed during the current study are available through the study coordinator, Seani Adrinah Mulondo (seani.mulondo@univen.ac.za) at the University of Venda on reasonable request.
